# First Complete Genome Sequence of *Suakwa aphid-borne yellows virus* from East Timor

**DOI:** 10.1128/genomeA.00718-16

**Published:** 2016-07-28

**Authors:** Solomon Maina, Owain R. Edwards, Luis de Almeida, Abel Ximenes, Roger A. C. Jones

**Affiliations:** a School of Plant Biology and Institute of Agriculture, University of Western Australia, Crawley, Western Australia, Australia; b Cooperative Research Centre for Plant Biosecurity, Canberra, Australia; c CSIRO Entomology, Floreat Park, Western Australia, Australia; d Seeds of Life Project, Ministry Agriculture and Fisheries, Dili, East Timor; e DNQB-Plant Quarantine International Airport Nicolau Lobato Comoro, Dili, East Timor; f Department of Agriculture and Food Western Australia, South Perth, Western Australia, Australia

## Abstract

We present here the first complete genomic RNA sequence of the polerovirus *Suakwa aphid-borne yellows virus* (SABYV), from East Timor. The isolate sequenced came from a virus-infected pumpkin plant. The East Timorese genome had a nucleotide identity of 86.5% with the only other SABYV genome available, which is from Taiwan.

## GENOME ANNOUNCEMENT

To examine possible connectivity between viruses infecting crops in northern Australia and nearby Southeast Asian countries, cucurbit samples from East Timor and Australia were studied. In July 2015, 15 leaf samples were collected from cucurbit plants in East Timor and blotted onto Fast Technology for nucleic acids (FTA) cards. In October 2015, 21 leaf samples were collected from cucurbit plants growing in the Broome district of the West Kimberley region of tropical northwest Australia. RNA extracts from 15 East Timorese and 21 Australian samples were subjected to next-generation sequencing. A complete genome of *Suakwa aphid-borne yellows virus* (SABYV) was obtained from a single pumpkin plant sample (DL76) from the Dili district of East Timor, but no SABYV was detected in any of the Australian samples. SABYV is a single-stranded RNA virus in the genus *Polerovirus*, family *Luteoviridae*. It causes leaf interveinal yellowing in cucurbit plants and occurs in the Philippines and Thailand in Southeast Asia, and in China and Taiwan in East Asia (1–3). Currently, there is only one complete SABYV genome in GenBank (accession no. JQ700308 from Taiwan) (4).

Total RNA was extracted from the samples using a ZR Plant RNA MiniPrep kit (Zymo Research), treated with RNase-free DNase (Invitrogen) and measured using Qubit (Invitrogen). RNA integrity was confirmed using RNA screen Tape (TapeStation 2200, Agilent Technologies). Libraries were prepared from total RNA extracts using a TruSeq Stranded Total RNA sample preparation kit with Ribo-Zero Plant (catalogue no. RS-122-2401, Illumina). Final size and concentration of each library was verified using Qubit and D1000 ScreenTape (TapeStation 2200). Sequencing was done with the MiSeq platform reagent kit version 2 (Illumina) using 2 × 251 paired-end runs with a 1% PhiX spike. Reads were assembled and genomes annotated using CLC Genomics Workbench (CLC Bio) and Geneious version 8.1.7 (Biomatters) (5); further alignment was done by MAFFT (6).

FTA card sample DL76 yielded 788,236 reads and, after trimming, 670,062 remained. *De novo* assembly generated 11 contigs; 13,045 reads were mapped to the contig of interest, giving a coverage of 330×. The assembled full-length genome from DL76 was 5,845 nucleotides in length with the six open reading frames typical of the genus *Polerovirus*. A BLAST-based search through the Pairwise Sequence Comparison (PASC) tool (7) revealed an isolate resembling Taiwanese isolate TW19. Comparison of their genomic sequences found that East Timorese isolate DL76 had a pairwise nucleotide identity of 86.5% to JQ700308. There is a need for further sampling to establish whether SABYV has spread to Australia. Comparison of any Australian SABYV genomic sequences found with ones from neighboring Southeast Asian countries would then be required.

### Nucleotide sequence accession number.

The new sequence was deposited in GenBank under the accession number KX122023 (DL76).
